# Intrinsically Disordered and Pliable Starmaker-Like Protein from Medaka (*Oryzias latipes*) Controls the Formation of Calcium Carbonate Crystals

**DOI:** 10.1371/journal.pone.0114308

**Published:** 2014-12-09

**Authors:** Mirosława Różycka, Magdalena Wojtas, Michał Jakób, Christian Stigloher, Mikołaj Grzeszkowiak, Maciej Mazur, Andrzej Ożyhar

**Affiliations:** 1 Department of Biochemistry, Faculty of Chemistry, Wrocław University of Technology, Wrocław, Poland; 2 Division of Electron Microscopy, Biocenter, University of Würzburg, Würzburg, Germany; 3 NanoBioMedical Centre and Department of Macromolecular Physics, Faculty of Physics, Adam Mickiewicz University, Poznań, Poland; 4 Department of Chemistry, University of Warsaw, Warsaw, Poland; Russian Academy of Sciences, Institute for Biological Instrumentation, Russian Federation

## Abstract

Fish otoliths, biominerals composed of calcium carbonate with a small amount of organic matrix, are involved in the functioning of the inner ear. Starmaker (Stm) from zebrafish (*Danio rerio*) was the first protein found to be capable of controlling the formation of otoliths. Recently, a gene was identified encoding the Starmaker-like (Stm-l) protein from medaka (*Oryzias latipes*), a putative homologue of Stm and human dentine sialophosphoprotein. Although there is no sequence similarity between Stm-l and Stm, Stm-l was suggested to be involved in the biomineralization of otoliths, as had been observed for Stm even before. The molecular properties and functioning of Stm-l as a putative regulatory protein in otolith formation have not been characterized yet. A comprehensive biochemical and biophysical analysis of recombinant Stm-l, along with *in silico* examinations, indicated that Stm-l exhibits properties of a coil-like intrinsically disordered protein. Stm-l possesses an elongated and pliable structure that is able to adopt a more ordered and rigid conformation under the influence of different factors. An *in vitro* assay of the biomineralization activity of Stm-l indicated that Stm-l affected the size, shape and number of calcium carbonate crystals. The functional significance of intrinsically disordered properties of Stm-l and the possible role of this protein in controlling the formation of calcium carbonate crystals is discussed.

## Introduction

Otoliths in teleost fish and otoconia in mammals are involved in the function of the inner ear, the sensory organ that plays an important role in hearing and balance [1], [2]. Several human disorders are associated with otoconia deficiency, dislocation, malformation, or age-related degeneration [3]–[5]. Both otoliths and otoconia are composed of calcium carbonate with a small amount of organic matrix composed of a mixture of different macromolecular components, including proteins, saccharides, glycans, and lipids [6]. These components act as a template for crystal nucleation and modulate crystal growth in desired directions [1], [2], [7]. Although the presence of otoliths or otoconia is conserved in all vertebrates, their developmental origins, growth, and the role of the matrix, especially the protein component, are still poorly understood. Thus, an investigation of the structural features of specific proteins which control crystal growth is an important step to help in understanding, and possibly imitating, the process of calcium carbonate biomineralization. It has been shown that many organic matrix proteins are extremely acidic and have extensive post-translational modifications [8]. Moreover, they often belong to the family of intrinsically disordered proteins (IDPs) [9], a class of proteins devoid of a rigid tertiary structure [10]–[12].

Starmaker (Stm) from zebrafish (*Danio rerio*) was the first protein found to be capable of controlling the process of calcium carbonate biomineralization in otoliths [13]. Stm is a functional analog of human dentin sialophosphoprotein (DSPP) which is a key factor required for proper teeth formation and is also expressed in the human ear [3], [14]. Stm is a highly acidic protein with many putative phosphorylation sites and potential calcium binding ability, thus it is considered to be a member of the secretory calcium-binding phosphoprotein family [15]. Stm controls the size, shape, and polymorph of the mineral component of the otolith and is, therefore, a key regulator of otolithic biomineralization [13]. As calcium-binding phosphoproteins are poorly evolutionarily conserved, the identification and characterization of other family members is a challenge [16]. Recently, the *starmaker-like* (*stm-l*) gene was identified in a large-scale random *in situ* hybridization screen of genes expressed in the developing ear of the medaka (*Oryzias latipes*). Its genomic structure and expression pattern are highly similar to the *starmaker* (*stm*) gene [15]. Although there is no obvious sequence similarity between *stm-l* and *stm* encoded proteins (Stm-l and Stm, respectively) and Stm has a significantly higher molecular weight than Stm-l (64.0 kDa for Stm without a signal peptide, and 39.4 kDa for Stm-l), it has been suggested that Stm-l could be involved in the biomineralization of otoliths as had been earlier observed for Stm [13]. Just as the human *dspp* gene (encoding DSPP), *stm-l* is also expressed in fry teeth [15], which might suggest a common origin. However, the protein encoded by the *stm-l* gene has not been characterized yet.

To facilitate exploration of the molecular basis of the Stm-l protein function, we have elaborated and optimized a protocol for the efficient expression and purification of homogeneous, non-tagged Stm-l. We present comprehensive biochemical and biophysical characteristics of the Stm-l protein along with *in silico* examinations which indicated, despite the fact that the Stm-l primary structure is different than Stm, that Stm-l exhibits properties typical of a coil-like IDP. Moreover, Stm-l possesses a highly pliable structure which can be easily modulated by external factors. Temperature or TFE (2,2,2-trifluoroethanol) caused the formation of ordered secondary structures in Stm-l. On the other hand, small concentrations of GdmCl (guanidine hydrochloride) and counter ions resulted in the compaction of the protein, which did not correlate with an increase in the content of secondary structures. *In vitro* experiments demonstrated that Stm-l controlled the size, shape and number of calcium carbonate crystals. The pliability of Stm-l, which leads to the formation of a more compact protein structure, may play a crucial role in the biomineralization of calcium carbonate. The possible roles of the structural features of Stm-l and how they function in the biomineralization process are discussed in the article.

## Materials and Methods

### Buffers

All buffers were prepared at 24°C. Buffer A was 20 mM Tris, 150 mM NaCl, pH 7.5. Buffer B was 20 mM Tris, 1 M NaCl, pH 7.5. Buffer L was 20 mM Tris, 150 mM NaCl, 1 mM dithiothreitol (DTT), 0.2 mg/ml phenylmethylsulfonyl fluoride (PMSF), pH 7.5.

### Construction of the expression vector

The sequence of the full-length Stm-l protein, the putative product of the *stm-l* gene, was taken from the Protein database (GenBank: ACR78440.1). cDNA of Stm-l was de novo synthesized in GeneArt® (Life Technologies, USA) and the gene sequence was optimized using GeneOptimizer® software to maximize the expression of the synthetic gene in *Escherichia coli*. Optimized full-length Stm-l cDNA was amplified using PCR [17] and the following primers: 5′- GCCGCGGGATCCatgaaaaattccgatgatgaaagc-3′ as a forward primer and 5′- GCCGCGAAGCTT *TCA*cattgctgcatctgccggcg-3′ as a reverse primer, containing the *Bam*HI and *Hind*III restriction enzyme sites, respectively (underlined). The small letters in the primer sequences indicate sequences originating from Stm-l optimized cDNA, whereas capitalized letters indicate nucleotides added for cloning purposes. Italic letters represent the stop codon on the reverse primer. The PCR product was purified using a Clean-Up kit (A&A Biotechnology, Poland), double-digested by *Bam*HI and *Hind*III endonucleases and cloned into corresponding sites of the pQE-80L (Qiagen, Germany) vector derivative obtained in our laboratory without the His-tag. The presence of the insert within the vector was confirmed by restriction analysis (data not shown) and the purified construct was verified by DNA sequencing. The final expression product designated Stm-l, had MGS amino acids added to the N-terminus.

### Overexpression and purification of recombinant Stm-l

The final plasmid construct containing the optimized Stm-l cDNA sequence was transformed into competent cells of *E. coli* strain BL21(DE3)pLysS (Novagen, Germany). A single colony was amplified in 50 mL of TB (Terrific Broth) media containing 50 µg/mL carbenicillin and 35 µg/mL chloramphenicol, in an incubator at 29°C at 220 rpm. Cell aliquots (3% of the total culture volume) were then used to inoculate larger volume cultures. When the OD_600_ of the culture reached a value of 0.8–0.9, the protein expression was induced by adding isopropyl-β-d-thiogalactopyranoside (IPTG) to the final concentration of 0.25 mM. After 3 h of incubation, bacterial cells were harvested by centrifugation at 4000 rpm using an Eppendorf A-4-81 rotor (Eppendorf AG, Germany) for 20 min at 4°C. The resulting cell pellet was washed with buffer L (24 mL per 1 L of bacterial culture), centrifuged at 4000 rpm using an Eppendorf A-4-81 rotor for 20 min at 4°C, resuspended in buffer L (24 mL per 1 L of bacterial culture), and finally divided into 50 mL Falcon tubes (600 mL of bacterial culture per each) and stored at −80°C.

The frozen cells from a total of 1.2 L of culture, were disrupted by quick thawing in a 24°C water bath, refrozen at −80°C, thawed again at 24°C and placed on ice. Then, DNase I and RNase A were added to the final concentration of 20 µg/mL of each enzyme and the lysates were incubated on ice until the bacterial nucleic acids were completely digested. The cell extract was then clarified by centrifugation at 12 000 rpm using an Eppendorf F-34-6-38 rotor for 40 min at 4°C, and the supernatant was fractionated on ice by salting out using solid (NH_4_)_2_SO_4_ (55% to 75% saturation). The pellet was immediately dissolved in 6 mL of buffer A and dialyzed overnight with buffer A (3×250 mL). The resulting solution was concentrated to a volume of 1 mL using the Amicon Ultracel-4 Centrifugal Filter Unit (Merck Millipore, USA) with a cut-off limit of 30 kDa and injected to the HiLoad 16/600 Superdex 200 prep grade column (Amersham Bioscience, UK) equilibrated with buffer A. The column was operated at room temperature at a 0.8 mL/min flow rate on the ÄKTAexplorer system (Amersham Bioscience, UK). Fractions containing Stm-l (ca. 15 mL) were combined and applied to the MonoQ 5/50 GL column (Amersham Bioscience, UK) equilibrated with buffer A and connected to the ÄKTAexplorer system. We experimentally elaborated the linear gradient of NaCl (150–500 mM, using buffer B) at a 1 mL/min flow rate applied for 20 min at room temperature. Fractions containing purified Stm-l were combined, concentrated to a volume of 200 µL using the Amicon Ultracel-4 Centrifugal Filter Unit with a cut-off limit of 10 kDa and desalted using a HiTrap Desalting column (Amersham Bioscience, UK) equilibrated with buffer A and connected to the ÄKTAexplorer system. All Stm-l fractions were combined, divided into small volumes (25 µL, 50 µL and 100 µL) and stored at −80°C.

The protein concentration was determined spectrophotometrically at 280 nm. The absorption coefficient for Stm-l was 0.139 mL/(mg × cm), calculated according to the method proposed by Gill and von Hippel [18]. For the *in vitro* calcium carbonate mineralization assay, the concentration of purified protein was determined using the biuret method [19].

### In silico analysis

The amino acid composition was analyzed using the Composition Profiler [20] available on the web page http://www.cprofiler.org. Analysis of disordered regions was carried out using PONDR-VLXT [21], available at http://www.pondr.com, PONDR-FIT [22] at http://www.disprot.org/pondr-fit.php, DISOPRED2 [23] at http://bioinf.cs.ucl.ac.uk, FoldIndex [24] at http://bip.weizmann.ac.il/fldbin/findex, IUPred [25] at http://iupred.enzim.hu, GlobPlot 2.3 [26] at http://globplot.embl.de. The prediction for secondary structures was performed using the network protein sequence server (NPS) [27] available at http://npsa-pbil.ibcp.fr. Charge-hydropathy plot [12] calculations were made using the PONDR server (http://www.pondr.com).

### SDS-PAGE gel electrophoresis

12% SDS-PAGE gels (1.0 mm thickness) were prepared according to Laemmli [28]. Electrophoresis was performed at a constant current of 20 mA/gel. After protein separation, gels were stained with either Coomassie Brilliant Blue R 250 [29] or carbocyanine dye (Stains-All; Sigma, Poland) [30], [31]. An Unstained Protein Molecular Weight Marker was used (Thermo Fisher Scientific Inc., USA).

### ESI mass spectrometry

Purified Stm-l (30 µg) was desalted using a PepRPC HR 5/5 column (Amersham Bioscience, UK) connected to the ÄKTAexplorer system, equilibrated with 0.05% trifluoroacetic acid (TFA). The linear gradient of acetonitrile (0–70%) at a 1 mL/min flow rate was applied for 30 min at room temperature. High-resolution mass spectrometry was performed using the microTOF-GTM spectrometer (Bruker Daltonik GmBH, Germany), equipped with an Apollo II electrospray ionization source with a ion funnel. The protein solution was infused at a flow rate of 3 µL/min. The mass spectrometer was operated in the positive ion mode. The mass resolution was 15 000 FWHM. The instrument parameters were as follows: a scan range of m/z 300–2300, the dry gas was nitrogen, and at a temperature of 180°C. Before performing each measurement the instrument was externally calibrated with the Tunemix mixture (Bruker Daltonik GmBH, Germany) in the quadratic regression mode.

### Analytical ultracentrifugation

Analytical ultracentrifugation (AUC) sedimentation velocity (SV) experiments were performed at 20°C on a Beckman XL-I analytical ultracentrifuge (Beckman Coulter Inc., USA) in an An-60 Ti rotor. Detection of the protein concentration as a function of radial position and time was performed by optical density measurements at a wavelength of 280 nm. Stm-l samples (400 µL in a concentration of 2.50 mg/mL, 1.22 mg/mL, or 0.40 mg/mL in buffer A) were loaded in 1.2 mm-thick two-channel centerpieces and centrifuged at 40 000 rpm. Data were analyzed with SEDFIT software using a continuous size distribution c(s) model to extract the sedimentation coefficients, s [32]. These s-values, after correction for solvent density and viscosity in relation to the density and viscosity of water at 20°C, were expressed as s_20,w_. Stm-l dimensions (Stokes radius, R_S_ and molecular weight, MW) were calculated by SEDFIT [32].

### Analytical size-exclusion chromatography

Analytical size-exclusion chromatography (SEC) was performed using a Superdex 200 10/300 GL column (Amersham Bioscience, UK) connected to the ÄKTAexplorer system, equilibrated with buffer A at room temperature with a flow rate of 0.5 ml/min. Purified Stm-l in three different concentrations (0.1 mg/mL, 0.5 mg/mL, or 2.5 mg/mL) were loaded in a total volume of 0.2 mL. Detection was achieved by monitoring the UV absorbance at 280 nm and 220 nm. The column was calibrated using the following standard proteins: thyroglobulin, 85.0 Å [33], apoferritin, 67.0 Å [34], bovine serum albumin, 35.5 Å [33], ovalbumin, 30.5 Å [33], chymotrypsinogen A, 20.9 Å[33], myoglobin, 20.2 Å [35], and cytochrome c, 17.0 Å [35]. The elution volumes for each standard and sample were measured and used to calculate the partition coefficients, K_av_ [36]. K_av_ for each standard was plotted against the corresponding R_S_ to generate a standard curve which was used to determine the approximate radius of Stm-l.

To perform SEC in the presence of GdmCl or counter ions, purified Stm-l was dissolved to a final concentration of 0.5 mg/mL in buffer A, supplemented with an appropriate concentration of GdmCl or counter ions in a total volume of 0.2 mL and incubated for 1 h at room temperature. The column was equilibrated with buffer A that contained the same amount of GdmCl or counter ions as the loaded sample. The previously calculated standard curve (see above) was used to estimate the R_S_ of Stm-l under all conditions [35]. Experiments with calcium and magnesium ions in the absence of sodium chloride were done as described above using 20 mM Tris pH 7.5 (24°C) instead of buffer A.

### Circular dichroism spectroscopy

Circular dichroism (CD) measurements were performed with a Jasco J-815 spectropolarimeter (Jasco Inc, USA) equipped with the Jasco Peltier-type temperature controller (CDF-426S/15) using quartz cuvettes with a path length of 0.1 cm in a spectral range of 190–260 nm. The Stm-l concentration was 10 µM. The study of intact protein was carried out in buffer A, whereas other measurements were performed after 1 h incubation at room temperature in the same buffer supplemented with appropriate concentrations of guanidine hydrochloride (GdmCl), trimethylamine N-oxide (TMAO), 2,2,2-trifluoroethanol (TFE) or calcium ions (CaCl_2_). In the experiment without sodium chloride, buffer A was changed to 20 mM Tris pH 7.5 (24°C) using the Amicon Ultracel-4 Centrifugal Filter Unit with a cut-off limit of 10 kDa and then supplemented with CaCl_2_. Spectra were collected (an average of 5 spectra) at a scanning speed of 20 nm/min at 20°C (for solution-induced protein folding/unfolding) and 50 nm/min at temperatures ranging from 20°C to 80°C and backward at 10°C intervals (for thermal-induced protein denaturation). Temperature-dependent denaturation was also monitored by following the changes in ellipticity at 222 nm, 208 nm, and 200 nm by increasing the temperature from 20°C to 80°C and then decreasing it from 80°C to 20°C at a constant rate of 1°C/min. All spectra were corrected for the effect of the respective buffers and converted to molar residual ellipticity units on the basis of the molar molecular mass per residue of 108.87 for Stm-l [37]. The analysis of secondary structure content was performed with deconvolution software CDPro [38] using IBasis 7 (SDP48) as the reference protein data set.

### Fluorescence Measurements

Fluorescence measurements were performed at 24°C using the Fluorolog-3 spectrofluorometer (HORIBA Jobin Yvon Inc., France). Intrinsic tryptophan fluorescence spectra of Stm-l (5 µM) were recorded with an excitation wavelength of 280 nm, and then fluorescence spectra were taken between 300 and 400 nm. A 3 mm path-length quartz cuvette 105.251-QS (Hellma GmbH & Co. KG, Germany) was used. In the experiment without sodium chloride, buffer A was changed to 20 mM Tris pH 7.5 using the Amicon Ultracel-4 Centrifugal Filter Unit with a cut-off limit of 10 kDa. The collected data were corrected for the contribution of the buffers.

### In vitro calcium carbonate mineralization assay

Calcium carbonate mineralization was performed using a slight modification of the technique described by Addadi et al. [39], which had been previously used in calcium carbonate mineralization in the presence of Stm [40]. This method is based on the decomposition of (NH_4_)_2_CO_3_ or NH_4_HCO_3_ into CO_2_ and NH_3_ and on slow carbon dioxide diffusion into the solution which contains calcium dichloride. We used Nunc MicroWel 96-Well Microplates (Thermo Fisher Scientific Inc., USA) with a circle cover glass (Waldemar Knittel Glasbearbeitungs GmbH, Germany) and 5 mm diameter on the bottom of each well. Thirty microliters of protein (final concentrations of 1, 5, 10, 20, and 50 µg/mL in buffer A) were incubated with CaCl_2_ (final concentrations of 5, 10, and 20 mM in Milli Q grade water) in a total volume of 0.3 mL per well. Control experiments were performed under the same conditions, except that either buffer A or trypsin (diluted in buffer A) in a concentration of 100 µg/mL was added instead of Stm-l. Two grams of solid ammonium hydrocarbonate (used instead of ammonium carbonate to minimalize the final concentration of ammonia) was covered with Parafilm (Bemis Company Inc., USA) with several holes (to slow down the diffusion) and placed above the 96-well plate in a closed desiccator. After 48 or 336 hours incubation at room temperature, the crystallization solution was removed and the crystals were washed with water and 96% ethanol and finally air-dried at room temperature.

### Scanning electron microscopy

The structural surface characterization of the calcium carbonate crystals was made by scanning electron microscopy (SEM) using a JEOL JSM-7500F scanning microscope (JEOL Ltd., Japan) at 5.0 kV. Each cover glass, with crystals on it, had been previously coated approx. 10–20 nm with gold/palladium (80/20) in argon gas using a BAL-TEC SCD 005 Sputter Coater (Leica Mikrosysteme Vertrieb GmbH, Germany). The size of the calcium carbonate crystals was determined from SEM images by measuring crystals for the edge length of parallele-piped crystals or the diameter of rounded crystals.

### Micro-Raman microscopy

Micro-Raman analysis of the calcium carbonate crystals was performed with a LabRAM 800 HR Raman confocal microscope (Horiba Jobin Yvon, Japan) equipped with a LPF Iridia edge filter, a 600 groove mm^−1^ holographic grating and a 1024×256 pixel Peltier-cooled Synapse CCD detector. The microscope attachment was based on an Olympus BX41 system with a MPLN50x objective and a motorized software-controlled x-y-z stage. The excitation source was the Excelsior-532-100 laser (Spectra-Physics, USA) operating at 523 nm with ca. 2 mW power on the sample. The Raman maps were recorded at 5 s integration time with 1 µm×1 µm spatial resolution. The Raman spectra were collected in the range of 50–4000 cm^−1^ (each spectral profile is an average of 5 spectra).

## Results

### Expression, purification and in silico analyses of Stm-l

**Figure 1:**
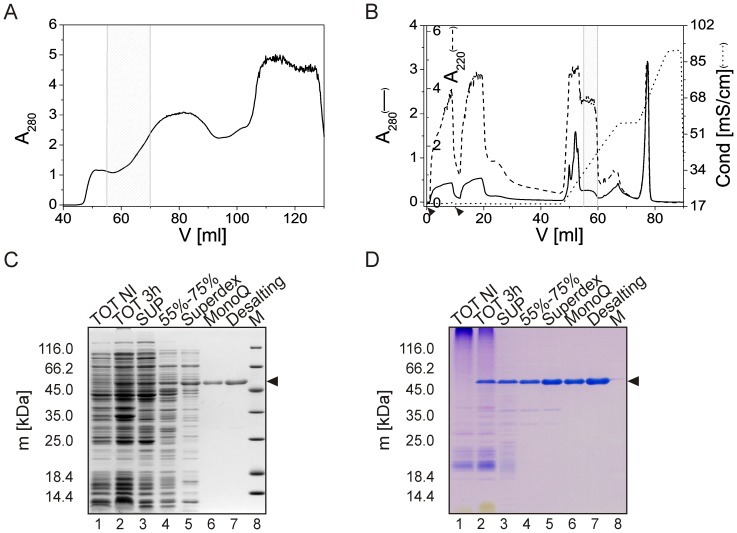
Purification of Stm-l. (A) Size exclusion chromatography using a HiLoad 16/600 Superdex 200 prep grade column. The absorption was measured at 280 nm. The hatched area on the chromatogram (elution volume 55–70 mL) indicates fractions which were combined and applied to the next step of the purification procedure. (B) Ion-exchange chromatography of Stm-l using a MonoQ 5/50 GL column. Fractions from gel filtration step containing Stm-l (ca. 15 mL) were combined and applied to the MonoQ 5/50 GL column. Because of the poor absorption of Stm-l at 280 nm, absorption was measured at 280 and additionally at 220 nm. The arrowheads indicate injection of the sample. Fractions containing purified Stm-l (hatched area) were combined, concentrated and desalted using a HiTrap Desalting column. (C) Commassie Brilliant Blue R 250-stained SDS-PAGE of the samples from the expression and purification of Stm-l. Lanes 1 and 2, the whole bacterial cell extract before and 3 h after the induction of the overexpression of Stm-l with IPTG, respectively; lane 3, the fraction of soluble proteins obtained after cell lysis; lane 4, proteins from a 55–75% ammonium sulfate fraction; lane 5, the combined gel filtration fractions; lane 6, the combined fractions after ion-exchange chromatography; lane 7, purified Stm-l after desalting using a HiTrap Desalting column; lane 8, molecular mass standards. The arrowheads in (C) and (D) mark the positions of Stm-l. (D) Analogical analysis as in (C), but the gel was stained using Stains-all dye [31].

To facilitate exploration of the molecular basis of the Stm-l function, we have elaborated and optimized a protocol for the efficient expression and purification of non-tagged Stm-l. The non-tagged Stm-l was overexpressed in an *E. coli* BL21(DE3)pLysS strain from a pQE80L vector derivative encoding the optimized cDNA sequence of Stm-l. Initially, a purification protocol for Stm without a signal peptide was used in the experiments [41], but unfortunately, because of the different molecular properties of Stm-l, it was not possible to obtain a homogenous protein sample (data not shown). Thus, a new purification method for obtaining a good yield of pure protein was elaborated. Small-scale experiments revealed that Stm-l is soluble in a fraction of ammonium sulfate up to 55%, whereas increasing the saturation to 75% led to the complete precipitation of Stm-l (data not shown). Thus, we decided to use a 55–75% ammonium sulfate saturation to precipitate Stm-l. Preliminary size-exclusion chromatography (SEC) experiments showed that Stm-l had an elution volume that corresponded to a protein with a higher apparent molecular mass. SEC was then chosen as the next step of the purification procedure, which made it possible to separate Stm-l from the large amount of contaminants present in the ammonium sulfate fraction (Fig. 1A). Ion-exchange chromatography with a MonoQ column was a key step in the Stm-l purification method (Fig. 1B). Because of the high content of amino acids with acidic side chains (calculated pI = 3.81), Stm-l was sufficiently attached to the column to separate it from the remaining contaminants including the degradation products (Fig. 1C, and D, lane 6). In the final step of the Stm-l purification procedure the sample was desalted using a HiTrap Desalting column to obtain the protein in the buffer with a standard concentration of NaCl. Purified Stm-l appeared as a single band on the 12% SDS-PAGE gel (Fig. 1C, and D, lane 7). Because of the poor binding of the Coomassie Brilliant Blue R-250 dye to Stm-l because of its low hydrophobicity [42], we decided to also use a different dye to detect Stm-l. The high content of acidic amino acids in the Stm-l sequence enabled us to use Stains-All (carbocyanine) dye, which stained acidic and calcium-binding proteins blue, while all other proteins were stained red [31]. The sample was subjected to the electrospray ionization mass spectrometry to identify Stm-l. The resulting value of 39 520.0 Da (data not shown) differed from the expected value calculated with the ProtParam tool [43] (39 651.3 Da) by 131.3 Da. This was most probably caused by the specific digestion of the N-terminal formylmethionine [44]. The described purification procedure usually yielded up to 4 mg of Stm-l from 1.2 L of growth medium used for the cell culture.

Interestingly, SDS-PAGE analysis revealed some abnormal characteristics of Stm-l. The apparent molecular mass of the recombinant protein calculated by the SDS-PAGE electrophoretic mobility was 52 320±230 Da (Fig. 1C, and D, black arrowheads), which represents 132% of the value of the molecular mass of Stm-l obtained in the ESI mass spectrometry (39 520 Da). This is typical for members of the family of intrinsically disordered proteins (IDPs), which fail to form rigid 3D structures under physiological conditions *in vitro* [11]. Flexible and dynamic IDPs can have very different structural features, ranging from the collapsed, molten globule-like conformation to the extended, premolten globule-like conformation, or even the coil-like conformation [10], [45], [46]. The major functional advantages of these proteins are believed to be their structural plasticity and pliability originating from the lack of a definite, ordered 3D structure, and enabling them to interact with a broad range of binding partners including other proteins, membranes, nucleic acids and various small molecules [47], [48]. It was previously demonstrated that IDPs are involved in many important biological processes such as signal transmission, the regulation of cell cycles, the regulation of gene expression, the activity of chaperone proteins, neoplastic processes, and biomineral formation [9], [46], [49], [50]. Amino acid sequences and compositions of IDPs are very different from those of ordered globular proteins. Thus, IDPs bind less SDS molecules and the apparent molecular mass obtained from SDS-PAGE is often 1.2–1.8 times higher than the real one calculated from sequence data or measured by mass spectrometry [11].

**Figure 2:**
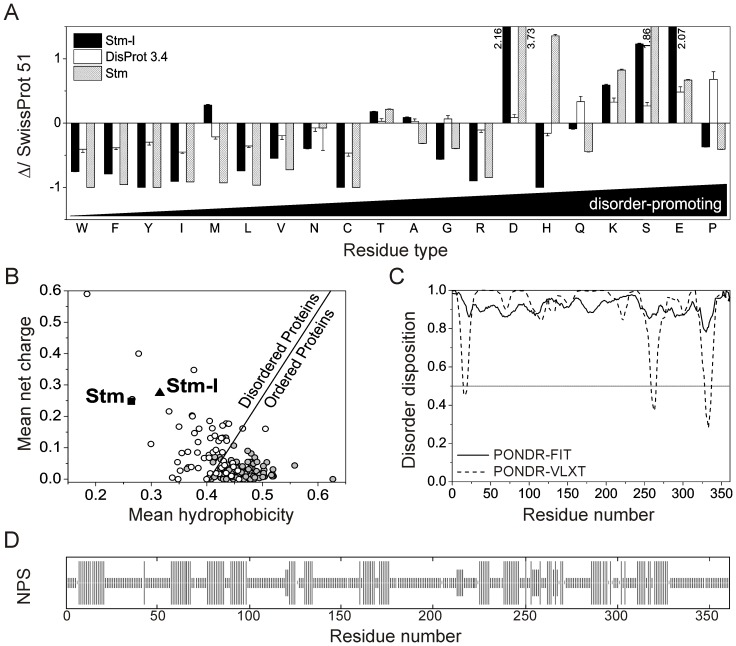
*In silico* analysis of the Stm-l sequence. (A) Amino acid composition. Composition Profiler [20] was used to analyze amino acid composition of Stm-l, IDRs represented by the DisProt 3.4 dataset [52], and Stm (black, white, and checked bars respectively). Values above zero indicate an abundance and values below zero indicate a deficiency of a given residue relative to the protein from the SwissProt 51 database. The amino acids are arranged from the most order-promoting on the left to the most disorder-promoting on the right according to the TOP-IDP scale [54]. (B) Charge-hydropathy analysis. The Uversky plot [12] compares the absolute, mean net charge and the mean hydropathy of 54 completely disordered proteins (open circles) and 105 completely ordered proteins (gray circles). The line represents the boundary between ordered and disordered proteins. The black triangle and square correspond to Stm-l and Stm respectively. (C) The prediction of disordered regions from an amino acid sequence. The prediction of a tendency for intrinsic disorder in Stm-l was calculated from its primary structure using different globular and disordered region predictors. PONDR-VLXT (dashed line) [21] is based on local amino acid composition, flexibility and other sequence features while PONDR-FIT (solid line) [22] combines the outputs of several individual disorder predictors. A score above 0.5 indicates a high probability of disorder. (D) The prediction of the degree of disorder. The plot represents the consensus predictions from 9 secondary structure predictors available within the NPS (Network Protein Sequence Analysis): SOPM, HNNC, MLRC, DPM, DSC, GOR I, GOR III, PHD, PREDATOR [27]. Each bar corresponds to one amino acid residue. The longest bars stand for α-helices, the medium bars represent β-structures and the shortest bars represent residues that were expected to form a random coil conformation.

The extremely high acidic character of Stm-l, its lowered electrophoretic mobility and the elution volume in the SEC that corresponded to an unusually high hydrodynamic radius, suggests that Stm-l belongs to the family of IDPs. It was previously demonstrated that proteins involved in biomineralization are frequently disordered. Intrinsic disorder was suggested as providing benefits to these proteins and enabling them to carry out their functions [9], [51]. This all inspired us to take a closer look at the molecular properties of Stm-l. The amino acid composition of Stm-l was first examined *in silico*. The Composition Profiler [20] web-based tool was used to identify statistically significant patterns of amino acid abundance (above zero) or depletion (below zero). Fig. 2A shows the composition profiles of Stm-l, Stm and the set of consensus sequences of the experimentally determined disordered regions represented by the DisProt 3.4 dataset [52] compared to the amino acid composition profiles found in nature that are listed in the SwissProt 51 dataset [53]. Amino acids are arranged from the most order-promoting on the left to the most disorder-promoting on the right, according to the TOP-IDP scale [54]. This analysis revealed that Stm-l has a distinctive distribution of amino acids. It is depleted in amino acid residues classified as order-promoting (W, F, I, L, V, and N), and two of them (Y, C) were completely absent in the protein. At the same time, the Stm-l sequence is rich in amino acid residues characterized as disorder-promoting (D, K, S, and E). According to the literature, this kind of amino acid distribution is typical of IDPs [10]. There were, however, some disorder promoting residues (G, H, Q, and P) that were clearly underrepresented in Stm-l. The high content of E and D residues (highlighted in bold in S1 Figure) could be responsible not only for the flexibility of Stm-l, but also for the extremely acidic character that is probably needed for it to function as a protein involved in calcium carbonate biomineralization. Surprisingly, the Stm-l sequence was rich in M residues, even compared to ordered proteins represented by the PDB S25 dataset (data not shown) [55]. The data presented in Fig. 2A indicated that the composition profile of Stm-l is very similar to the profile of Stm, which was classified as being a member of the IDPs [41]. Noteworthy is that both of these proteins have completely different primary structures [13], [15]. The molecular mass of Stm-l (39 651.3 Da) is almost two times smaller than Stm (64 628.2 Da) [41]. Moreover, in the Stm-l sequence there were no highly conserved internal repeats as was seen in the case of Stm [13]. The charge-hydropathy plot [12] showed that Stm-l is more hydrophobic than Stm and at the same time is more charged, although it can still be clearly classified as an IDP (Fig. 2B).

The putative intrinsic disorder propensities of Stm-l were also evaluated by an *in silico* analysis of the amino acid sequences using several different disorder predictors, such as PONDR-VLXT [21], PONDR-FIT [22], DISOPRED2 [23], FoldIndex [24], IUPred [25], GlobPlot 2.3 [26], and nine secondary structure predictors available within the network protein sequence (NPS) analysis [27]. Since the results of most of the predictors were compatible, we decided to show only three sets of results. The data clearly indicate that Stm-l is a highly disordered protein, although some potential regions of order were predicted by PONDR-VLXT and the NPS algorithm (Fig. 2C, and D).

Altogether, the unusual properties of Stm-l that were observed, including the unusually high hydrodynamic radius in SEC, the differences between the molecular mass value determined theoretically and by SDS-PAGE, the composition of the characteristic amino acid sequences and the predicted presence of disordered regions suggest that Stm-l exhibits the properties of an IDP.

### Hydrodynamic properties of Stm-l

**Figure 3:**
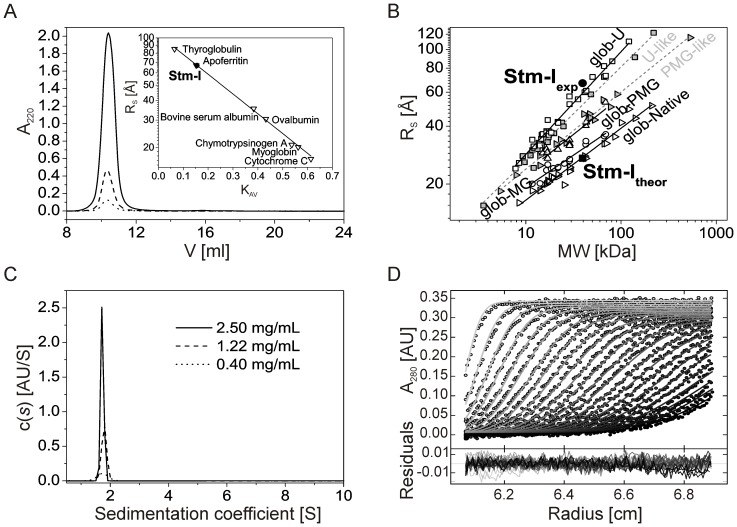
Hydrodynamic properties of Stm-l. (A) Analytical size-exclusion chromatography (SEC). Experiments were performed for three different protein concentrations: 0.1 mg/mL (dotted line), 0.5 mg/mL (dashed line), and 2.5 mg/mL (solid line). The inset shows the calibration curve with a black circle which corresponds to Stm-l. (B) Dependencies of the hydrodynamic radii (R_S_) to the relative molecular masses (MW) of four equilibrium states of globular proteins (solid lines) and for two subclasses of IDPs (dashed lines). The globular proteins are shown as open symbols: native (glob-Native, reverse triangles), molten globules (glob-MG, circles), pre-molten globules (glob-PMG, triangles), and 6 M GdmCl-unfolded proteins (glob-U, squares). The IDPs are shown as gray symbols: U-like (squares), and PMG-like (reverse triangles). The data for globular proteins were taken from [62] and for IDPs from [60]. Values for Stm-l are represented by black symbols: theoretical (square), and experimental (circle). (C) Sedimentation velocity (SV) analytical ultracentrifugation analysis. Superposition of the sedimentation coefficient distributions, c(s) derived via SEDFIT [32] from SV data for Stm-l in three different concentrations: 2.50 mg/mL (solid line), 1.22 mg/mL (dashed line), and 0.40 mg/mL (dotted line) measured at 280 nm during the SV experiment at 40 000 rpm at 20°C, standardized to water at 20°C. (D) Sedimentation profile of Stm-l. Superposition of selected experimental (circles) and fitted SV profiles (solid lines) corrected for all systematic noises for Stm-l at 2.50 mg/mL. An rmsd of 0.00493 indicates a good fit of the SV data. The inset shows the superposition of the differences between the experimental and fitted curves.

IDPs are known to have substantially larger hydrodynamic parameters compared to globular proteins [12], [56]. Thus, we decided to analyze this in detail for Stm-l. The hydrodynamic behavior of Stm-l was characterized by two independent methods: SEC and analytical ultracentrifugation (AUC). Both of these analytical techniques have been shown to be very useful in identifying IDPs [57]–[59]. SEC is the simplest method for analyzing hydrodynamic properties like the hydrodynamic radius of protein (Stokes radius, R_S_). The elution volumes (V_e_) of standard proteins were used to calculate the partition coefficients, K_av_ (Fig. 3A, inset) [36]. Interestingly, Stm-l was eluted from the Superdex 200 10/300 GL column with a V_e_ corresponding to the R_S_ 66.83±1.00 Å. This is significantly higher –2.45 times – than the 27.27 Å which was calculated from the sequence data assuming it is a globular protein (Table 1). The remarkably bigger R_S,exp_, in comparison to the theoretical value, placed the Stm-l on the R_S_ versus MW plot in the area for natively unfolded proteins with coil-like properties (U-like) (Fig. 3B) [12]. The apparent molecular mass value of 377 710±1000 Da was almost 10 times larger in comparison with the theoretical value (39 520.0 Da). These results together with the observed V_e_, which was independent of the sample concentration and had a single symmetric peak in all tested concentrations (Fig. 3A), suggests that Stm-l occurs as a monomer with a highly extended conformation [60].

**Table 1:** Identification of Stm-l by size exclusion chromatography.

	R_S_ [Å]		V_S_ ×10^−3^ [Å^3^]		ρ×10^5^ [kDa/Å^3^]	
MW [kDa]	theor^a^	exp^b^	theor^c^	exp^d^	theor^c^	exp^d^
39.5200	27.27	66.83±1.00	84.95	1250.27±56.02	46.52	3.16±0.14

a Determined from the equation: log (R_S_)  =  (0.395±0.016) log (MW) + (0.805±0.031).

b Determined from the equation: log (R_S_)  =  −(1.277±0.027) K_av_ + (2.022±0.012).

c Calculated using the theoretical R_S_.

d Calculated using the experimental R_S_.

To unequivocally determine whether Stm-l is an oligomer or an extended monomer, we decided to perform velocity sedimentation AUC experiments to estimate the shape of the protein as a complementary technique to the methods based on size determination (e.g., SEC) [57]. We analyzed three different sample concentrations: 0.40, 1.22, and 2.50 mg/mL (Fig. 3C, Table 2). Data analysis for all three concentrations yielded a very good fit with a rmsd of 0.0039, 0.0049 and 0.0069 (Fig. 3D). The computed sedimentation coefficients (s_20,w_) appeared to be independent of the sample concentration (Table 2). IDPs have been shown to have extended shapes with a large value of the frictional ratio (f/f_min_) which increases with the size of the IDP [61]. For example, the f/f_min_ is 2.1 for a 20-kDa and 3.0 for a 200-kDa coil-like IDPs, and 1.75 for a 20-kDa and 2.05 for a 200-kDa PMG-like IDPs. These f/f_min_ values are remarkably higher than those observed for globular proteins, where there is only a very slight increase in the f/f_min_ with increases in MW (1.19 and 1.25 for MWs of 20 and 200 kDa, respectively) [12], [62]. The f/f_min_ calculated for Stm-l was unusually high, ranging from 2.70 to 3.35, indicating that Stm-l is a coil-like IDP. The obtained Stokes radii for different protein concentrations (68.4 Å for 0.40 mg/mL and 74.5 Å for 1.22 mg/mL) are in good agreement with the SEC data (66.83±1.00). The AUC technique usually determines MW with good accuracy. However, there was a problem in calculating the MW of Stm-l at higher concentration (2.50 mg/mL). This may have been due to the high negative charge of the protein. During the experiment, electrostatic repulsion of the Stm-l molecules at the bottom of the chamber could have prevented the protein from being concentrated with high density. In order to analyze the protein in higher concentrations, all data obtained for a radius above 6.9 cm had to be cut off, which is usually not necessary for non-charged proteins. Based on the results obtained in the SEC and AUC experiments, Stm-l may be safely assumed to be a monomeric protein. This is supported by the calculated experimental MW (39 679 Da for 2.50 mg/mL), similar to the value obtained in ESI mass spectrometry (39 520.0 Da) when the f/f_min_ was fixed at 2.64.

**Table 2:** Identification of Stm-l by sedimentation velocity analytical ultracentrifugation.

			Main species				
Concentration (mg/mL)	rmsd	f/f_min_	s_20,w_ (S)	s (S)	%^a^	R_S_ [nm]	MW [Da]
0.40	0.00371	2.91	1.555	1.720	66.1	6.84	43 252
1.22	0.00650	3.35	1.598	1.784	99.3	7.45	48 485
2.50	0.00493	2.57	1.496	1.670	98.2	9.10	55 370
2.50		2.64					39 679^b^

a A percentages given with 100% for all types of species.

b MW value obtained when the f/f_min_ was fixed at 2.64.

In summary, the unusually high f/f_min_ and R_S_ of Stm-l and the fact that this protein exists as a monomer in its native state according to two independent research methods (SEC and AUC), indicate that Stm-l is a highly asymmetric and unfolded molecule that exhibits properties of coil-like IDP.

### Secondary structure analysis of Stm-l by far-UV CD spectra

**Figure 4:**
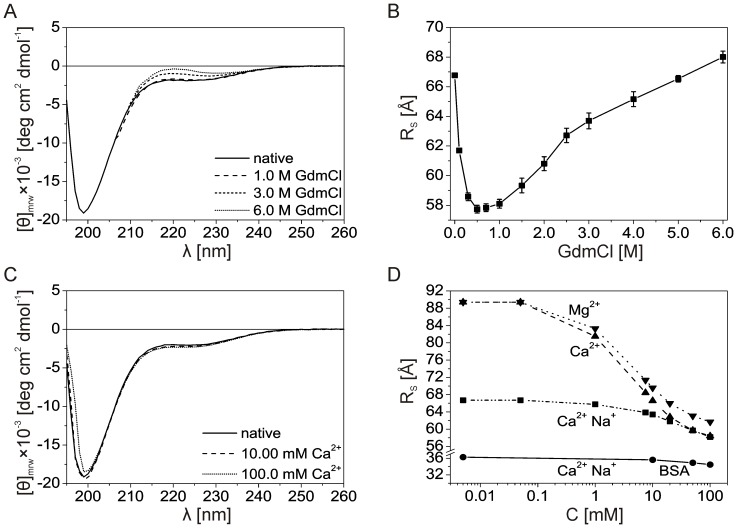
Conformational changes in Stm-l. (A) The far-UV CD spectra of Stm-l in denaturing conditions. Representative spectra recorded in buffer A at 20°C in the absence and presence of increasing concentrations of GdmCl. (B) SEC analysis of Stm-l in denaturing conditions. Using the calibration curve (Fig. 3A, inset), the R_S_ values of Stm-l were calculated and plotted versus the GdmCl concentration. (C) The effect of Ca^2+^ on the far-UV CD spectra of Stm-l in a buffer with 150 mM NaCl. Representative spectra recorded in buffer A at 20°C in the absence and presence of increasing concentrations of Ca^2+^. (D) Changes of the Stm-l R_S_ induced by calcium ions in the absence and presence of 150 mM NaCl estimated by a series of SEC analyses. Using the calibration curve (Fig. 3A, inset), the R_S_ values of Stm-l were calculated and plotted versus the ion concentration. Bovine serum albumin (BSA) was used as a control (shown on the plot as circles). Additional analysis of the effect of magnesium ions on the R_S_ of Stm-l was performed. Symbols for Stm-l are: squares, triangles and reversed triangles for Ca^2+^ with Na^+^, Ca^2+^ without Na^+^ and Mg^2+^ without Na^+^ ions, respectively.

Circular dichroism (CD) is a method for rapidly evaluating the secondary structure and folding properties of proteins. It has been demonstrated that different secondary structures in proteins exhibit distinctive far-UV CD spectra [63]. The CD spectrum of IDPs has a large negative peak at around 200 nm and a value close to zero at 222 nm, which is different from that of ordered conformations and make it possible to identify partially or fully disordered proteins [11]. The Stm-l spectrum is similar to spectra typical of other IDPs. Despite the fact that Stm-l displays the characteristic deep minimum at 200 nm ([θ]_200_ = −18.0×10^−3^ [deg × cm^2^ × dmol^−1^]), it also possesses a slight negative band at 222 nm ([θ]_222_ = −2.0×10^−3^ [deg × cm^2^ × dmol^−1^]), suggesting the existence of some residual ordered structures [64] (Fig. 4A). Analysis of the spectrum by CDPro spectra deconvolution software using SELCON3, CDSSTR, and CONTIN/LL programs with IBasis 7 [38] revealed that the dominant type of ordered structures in Stm-l are a β-strand (19.6±4.0%, with major contributions of a regular type: 12.0±2.5%) and turns (11.4±1.5%). Interestingly, Stm-l contains a very small amount of the α-helix (5.6±5.0%); however, the main part of Stm-l is unordered (62.7 5.5%; Table 3). A double-wavelength plot [θ]_222_ versus [θ]_200_ is used to classify IDPs into two structurally different groups: coil-like and PMG-like [60]. According to data obtained from the CD spectrum, Stm-l belongs to the group of proteins which are coil-like and which have almost no ordered secondary structure (data not shown). Data calculated from the CD spectra of Stm-l supports the observations obtained from the hydrodynamic properties analyses done by SEC and AUC techniques showing that Stm-l exhibits properties of a coil-like IDP.

**Table 3:** Determination of the structure of Stm-l by circular dichroism in the far-UV spectrum.

Agent		α-Helix (%)			β-Strand (%)			Turns (%)	Unordered (%)
		Regular	Distorted	Total	Regular	Distorted	Total		
	20°C	2.2±2.3	3.4±2.7	5.6±5.0	12.0±2.5	7.6±1.6	19.6±4.0	11.4±1.5	62.7±5.5
TFE	70%	12.8±0.4	11.5±1.4	24.3±1.4	8.0±0.5	6.0±0.4	14.0±0.9	15.1±2.0	45.7±4.5

The content of the secondary structure was calculated using CDPro spectra deconvolution software with IBasis 7 (SDP48). The results from SELCON3, CDSSTR, and CONTIN/LL were averaged and the standard deviation was calculated.

### Stm-l folding and unfolding

Protein folding and unfolding induced by various factors can provide information about protein conformation and compactness. IDPs have been shown to adopt a more ordered and rigid structure under the influence of different factors such as temperature, denaturants, osmolytes, binding partners, molecular crowding, or counter ions [65]. CD far-UV spectra in various solvents and temperatures [63], [66] were recorded to test whether Stm-l has properties similar to those of IDPs and can adopt a more ordered secondary structure in the presence of some of the factors mentioned above. The process of folding and unfolding was simultaneously monitored by SEC, since it is usually accompanied by changing hydrodynamic properties [67], [68].

Fig. 4A shows the representative spectra obtained for Stm-l in a buffer containing increasing concentrations of GdmCl. Because of the poor transparency of buffers with high concentrations of GdmCl, it was impossible to record CD spectra around 200 nm. Thus, the quantitative analysis of the whole spectra (in a spectral range of 190–260 nm) in denaturing conditions with CDPro software was not possible. Nevertheless, we performed analysis of the 222 nm signal change (data not shown). It is noteworthy that we observed relatively poor changes when GdmCl concentrations were equal or below 1 M. At high concentrations of the denaturant (3 M, and 6 M), however, ellipticity at 222 nm increased, reflecting the denaturation of ordered secondary structures. SEC in denaturing conditions was performed to gain additional information about the unfolding of Stm-l. The estimated R_S_ plotted against GdmCl concentrations showed that small amounts of denaturant (up to 0.5 M) caused a decrease in the R_S_ for Stm-l of 13.6% (from 66.8±0.1 Å in native conditions to 57.7±0.3 Å in 0.5 M GdmCl). An increase in GdmCl concentration led to the loosening of the previously compacted conformation and denaturation of Stm-l (Fig. 4B), showing a relatively small change in the R_S_ of 1.8%, from 66.8±0.1 Å in native conditions to 68.0±0.4 Å in 6 M GdmCl (Fig. 4B).

Because Stm-l was suggested as being a novel homolog of zebrafish Stm [15] which plays a crucial role in calcium carbonate mineralization [13], it is possible that calcium ions may influence its conformation, as was previously shown for Stm [59], [69]. Representative far-UV CD spectra in the absence and presence of increasing concentrations of calcium ions are shown in Fig. 4C. Analysis of the ellipticity at 222 nm indicates that calcium ions did not change the content of secondary structures in Stm-l (data not shown). However, a series of SEC experiments provided evidence that different concentrations of calcium ions could modulate the extended overall conformation of Stm-l. The plot of R_S_ as a function of divalent ion concentrations in a semi-logarithmic scale (Fig. 4D) confirmed that there was a gradual decrease in the R_S_ with increasing divalent ion concentration. Note that when a buffer was used which did not contain sodium chloride, the R_S_ of Stm-l significantly increased by 34.0% (66.8 Å in the presence and 89.4 Å in the absence of sodium chloride). The addition of 100 mM concentrations of calcium ions caused the Stm-l R_S_ to drop by about 12.9% (from 66.8 Å to 58.2 Å) when the buffer contained sodium chloride and by 34.7% (from 89.4 Å to 58.4 Å) in the absence of sodium chloride. BSA was used as a control and showed a very low degree of compaction with a change in dimension of only about 4.9% (from 36.3 Å to 34.5 Å) in the presence of NaCl. Although the presence of sodium chloride seemed to have had an influence on the Stm-l R_S_ in the absence of Ca^2+^, the addition of 50 mM of calcium ions eliminated the impact of sodium chloride on the compaction of Stm-l. Moreover, the far-UV CD spectra of Stm-l recorded in increasing concentrations of calcium ions in a buffer without sodium chloride was exactly the same as when a buffer with 150 mM NaCl was used. There was no noticeable impact from calcium ions on the content of the ordered secondary structure of Stm-l (data not shown). Interestingly, analysis of the W residue fluorescence emission spectrum revealed that the presence of NaCl did not affect the maximum fluorescence of Stm-l (data not shown). Both in the presence and in the absence of sodium chloride, the fluorescent emission spectrum of Stm-l, measured with an excitation of 280 nm had a maximum fluorescence of 359 nm, suggesting the presence of a W312 residue in a polar environment. However, this experiment cannot exclude the existence of a hydrophobic cluster. In conclusion, we have clearly shown, that calcium ions can modulate the extended conformation of Stm-l. Despite the fact that there was no impact on the formation of ordered secondary structures, calcium ions were found to cause the compaction of Stm-l conformation, depending on the presence of sodium chloride.

**Figure 5:**
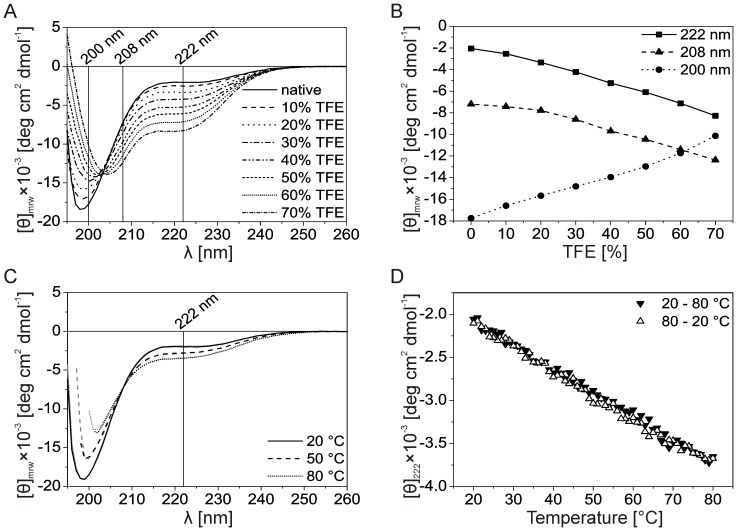
The effect of TFE and temperature on the secondary structure of Stm-l. (A) TFE-induces secondary structure formation in Stm-l. Representative spectra recorded in buffer A at 20°C in the absence and presence of increasing concentrations of TFE. (B) TFE-induces changes in the far-UV CD spectrum of Stm-l measured in the absence and presence of increasing concentrations of TFE. The linear plot showing [θ]_222_ (squares), [θ]_208_ (triangles), and [θ]_200_ (circles) as a function of the TFE concentration. (C) Temperature-induced secondary structure formation in Stm-l. Representative far-UV spectra of the protein measured at temperatures increased from 20°C to 80°C (black lines) and decreased (gray lines). (D) Temperature-induced changes in the far-UV CD spectrum of Stm-l ([θ]_222_ versus temperature dependence) measured at temperatures increased from 20°C to 80°C (black reversed triangles) and decreased (open triangles) with 1° intervals.

The majority of IDPs can form a more ordered structure upon binding to their targets. When the factors involved are unknown, the folding propensity of an IDP can be tested by using different agents which had been previously found to impact the conformation of IDPs [70], [71]. To test the structure-forming potential of Stm-l, we used 2,2,2-trifluoroethanol (TFE), which is known to increase the propensity of amino acids usually to form an α-helix [71]. Fig. 5A shows the impact of increasing TFE concentrations on the CD far-UV spectrum of Stm-l, and Fig. 5B shows changes in the ellipticity at 222 nm, 208 nm, and 200 nm as a function of the TFE concentration. The disappearance of the ellipticity minimum at 200 nm and the simultaneous appearance of two minima at 208 nm and 222 nm at increasing TFE concentrations, suggests that there was an increase in the content of secondary structures (Fig. 5A). Quantitative analysis of the spectra in the absence and presence of 70% TFE, computed using CDPro software, are shown in Table 3. At the highest concentration of TFE, the helical content increased by ca. 18.7%. At the same time, there was a decrease in the β-strand content by ca. 5.6%. This observation is consistent with previous reports which showed that TFE induced the transition of a β-strand to an α-helix [72], [73]. However, even at the highest concentration of TFE, the main part of Stm-l remained unordered (45.7%). Another agent used to assess the folding propensity of Stm-l was trimethylamine N-oxide (TMAO), the naturally occurring osmolyte [74] which induces folding not by binding but by solvophobic effects on the peptide backbone exposed in an unfolded state [70]. We recorded the far-UV CD spectra of Stm-l with increasing concentrations of TMAO up to 4.0 M, but the shape of all CD spectra were almost the same as the CD spectrum in the native condition (data not shown).

According to Uversky [65], analysis of the effect of temperature on the structural properties of IDPs characterized as native coils and native pre-molten globules revealed that they exhibit a so-called turned out response to heat, where increasing temperatures induce the formation of a secondary structure. Stm-l revealed similar properties as illustrated by Fig. 5, where the temperature-induced changes in the far-UV CD spectrum of Stm-l (Fig. 5C) and the temperature-dependence of the ellipticity at 222 nm (Fig. 5D) are shown. As the temperature increased, [θ]_222_ decreased, reflecting the temperature-induced formation of a secondary structure (Fig. 5D, black reversed triangles). Interestingly, changes in the [θ]_222_ versus temperature plot were linear, which means that the nature of folding was noncooperative. The structural heating-induced changes in Stm-l were completely reversible (Fig. 5D, open triangles). Because of the poor transparency of the buffer above 60°C, it was impossible to collect CD data around 200 nm. Thus, the quantitative analysis of the spectra at higher temperatures with CDPro software was not possible.

Altogether, the above results indicate that Stm-l is an IDP which has a pliable structure and a significant propensity for folding. The Stm-l structure is very sensitive to external factors and can be easily modulated by them, leading to the formation of ordered secondary structures or the compaction of Stm-l molecules.

### Stm-l changes the morphology of calcium carbonate crystals

It was previously suggested that Stm-l plays a biological role similar to Stm, which is required for the proper formation of otoliths. However, until now there has not been any experimental evidence to verify this hypothesis. An *in vitro* calcium carbonate mineralization assay was done to see if this hypothesis was true and to see if Stm-l had an impact on calcium carbonate mineralization.

**Figure 6:**
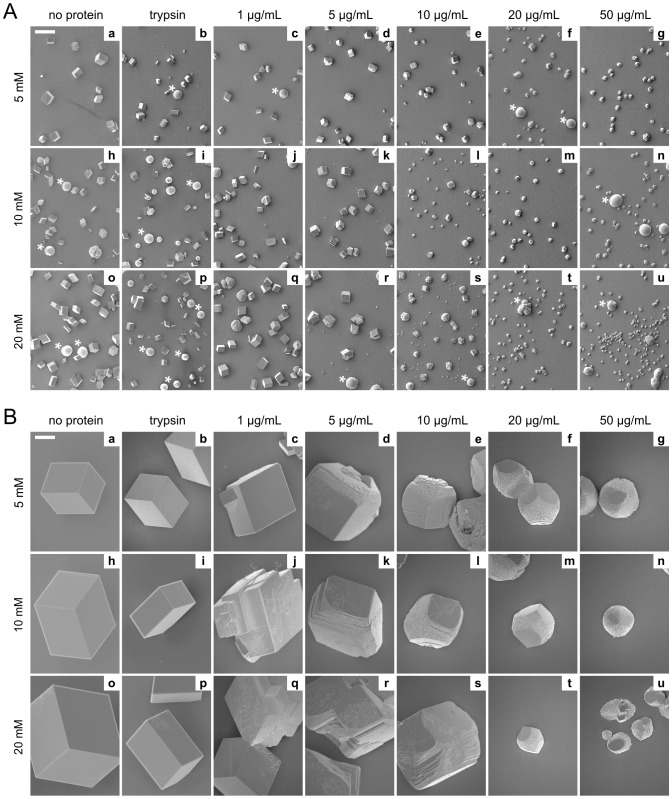
The effect of Stm-l on calcium carbonate mineralization. (A) SEM images of calcium carbonate crystals grown 48 h. Crystals grown in the absence of any protein (a, h, o), in the presence of trypsin at a concentration of 100 µg/mL (b, i, p), and in the presence of Stm-l at the following concentrations: 1 µg/mL (c, j, q), 5 µg/mL (d, k, r), 10 µg/mL (e, l, s), 20 µg/mL (f, m, t), 50 µg/mL (g, n, u). Concentrations of calcium ions were 5 mM (a–g), 10 mM (h–n), or 20 mM (o–u). Asterisks indicate additional, less thermodynamically stable, large spherical vaterite crystals which were present at every calcium ion and protein concentration and in the controls. The scale bar on the upper left corner of each panel represents a 200 µm-distance. (B) 10× magnification of the representative crystals shown on panel (A). The scale bar on the upper left corner of each panel represents a 20 µm-distance. Other details as in (A).

**Figure 7:**
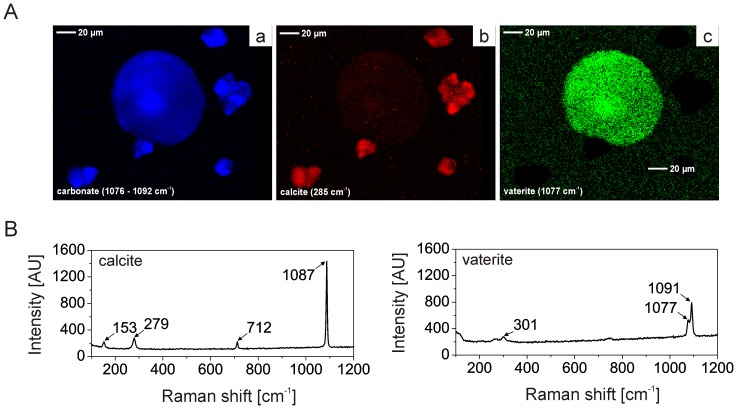
Micro-Raman analysis of calcium carbonate crystals grown in the presence of Stm-l. (A) Micro-Raman map of carbonate (a), calcite (b) and vaterite (c) for crystals obtained in the presence of Stm-l at a concentration of 50 µg/mL and 10 mM CaCl_2_ during mineralization period of 48 h. Asterisks indicate large spherical vaterite crystals (as in Fig. 6An), and arrowheads indicate smaller calcite crystals similar to this presented in Fig. 6Bn. (B) Micro-Raman spectra of calcite and vaterite of sample mapping in (A).

Fig. 6 shows the SEM morphologies of crystals grown in the presence of Stm-l in various concentrations, or in the presence of trypsin (as a control protein not involved in biomineralization), or without any protein during mineralization period of 48 h. Calcium carbonate crystals grown in the presence of Stm-l (Fig. 6A and B, images c–g, j–n, q–u) differed significantly in shape and size from the crystals obtained without any protein (Fig. 6A and B, images a, h, o), or in the presence of trypsin (Fig. 6A and B, images b, i, p). Noteworthy is the fact, that all the control crystals were prismatic. It was previously shown that synthetic calcite usually grows as an almost isotropic rhombohedron in the hexagonal lattice in which the calcium and carbonate ions are closely packed to obtain the most thermodynamic stability [75]. In contrast, crystals with Stm-l had a significantly different growth pattern which changed with increasing protein concentrations. The impact of Stm-l was already visible at a concentration of 1 µg/mL, when all the crystals became rounded at the edges (Fig. 6A and B, images c, j, q). However, a higher concentration of 5 µg/mL revealed crystals with noticeably rounded edges and characteristic stair-like structures (Fig. 6A and B, images d, k, r). At a protein concentration of 10 µg/mL – independent of the calcium ion concentration – we observed a heterogenic, two-sized population of crystals with significantly different dimensions (Fig. 6A and B, images e, l, s). Crystals were the most modified at a concentration of 20 µg/mL, when the edges and some of the faces of the crystals completely disappeared and the stair-like structures that had formed on the surface of the crystals were distinctly noticeable (Fig. 6A and B, images f, m, t). This effect was enhanced at the highest protein concentration of 50 µg/mL (Fig. 6A and B, images g, n, u). A higher protein concentration of 100 µg/mL was also tested, but the effect on the calcium carbonate mineralization was similar that for 50 µg/mL (data not shown). Micro-Raman analysis showed that crystals obtained in the presence of Stm-l even at the highest concentration of protein are calcite (Fig. 7). The same results was obtained for crystals obtained without any proteins or in the presence or trypsin (data not shown).

In addition to the crystals described above in the *in vitro* biomineralization assay, we observed rare crystals with a completely different morphology, which were present at any calcium ion and protein concentration and in the controls (Fig. 6A, marked with asterisks). They were characterized by a large size – much larger than the rest of the crystals – and a spherical shape with a small hole in the central part of the crystal. Interestingly, they were absent after a test period of 336 h, which means that they were less thermodynamically stable than other crystals (S2A Figure). Micro-Raman spectra showed, that these spherical deposits obtained in every test conditions are vaterite (Fig. 7).

**Figure 8:**
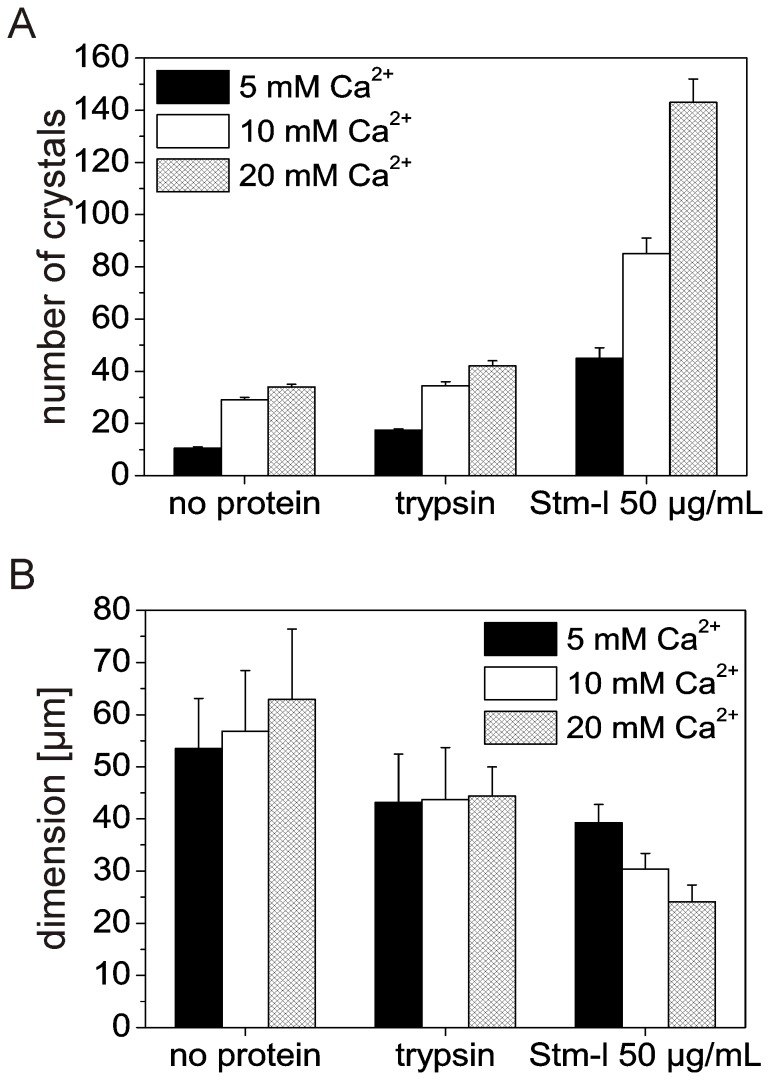
Analysis of the impact of Stm-l on the calcium carbonate formation process. (A) The number of calcium carbonate crystals was determined by counting them using 3 different SEM 1.2×0.9 mm images for each experimental conditions and then calculating the average number of crystals. (B) The average size of the calcium carbonate crystals was determined from SEM images by measuring crystals for their edge length or diameter, for prismatic and rounded crystals, respectively. Panels (A) and (B) show respective data obtained in the absence of any protein, in the presence of trypsin at a concentration of 100 µg/mL, and in the presence of Stm-l at a concentration of 50 µg/mL. Calcium ion concentrations were 5 mM, 10 mM, and 20 mM (black, white, and checked bars respectively).

Fig. 8 shows a comparison of the number and size of the crystals under determined different conditions in an experiment after 48 h of crystal formation. It is clear that Stm-l dramatically increased the number of crystals in comparison with the number of crystals obtained in the absence of the protein or in the presence of trypsin (Fig. 8A). The greatest increase in the number of crystals was seen for a calcium ion concentration of 20 mM. Stm-l also changed the dimension of the calcium carbonate crystals. The presence of 50 µg/mL of Stm-l decreased the size of crystals in comparison with crystals obtained in all control conditions with trypsin or without any protein (Fig. 8B). The smallest crystals were obtained in the highest calcium ion concentration of 20 mM. Moreover, there was a wide range in the crystal dimensions obtained for the negative control (without any protein) and for the control with trypsin, while the range of crystal sizes grown in the presence of the highest Stm-l concentration (50 µg/mL) was rather narrow. This suggests that at higher concentrations Stm-l inhibited crystal growth and maintained a uniform crystal size for given concentrations of calcium ions (Fig. 7B). Similar results were obtained with a mineralization period of 336 h; however, crystals were slightly larger than those obtained with an experimental period of 48 h (S2 Figure).

Altogether, these results indicate that Stm-l had an influence on the formation of calcium carbonate crystals. The increase in the number of calcium carbonate crystals and the decrease in their dimensions was depended on the concentration of Stm-l (compare images in Fig. 6). The nucleation of calcium carbonate crystals appeared to be controlled by Stm-l, acting as a template for crystal deposition. Stm-l also exerted an influence on the shape of growing crystals and simultaneously inhibited crystal growth, resulting in the formation of significantly smaller crystals with rounded edges.

## Discussion

Calcium carbonate biominerals are widespread in nature. In eukaryotes, all biologically controlled calcium carbonate minerals are associated with an organic matrix, which is involved in essential functions such as crystal nucleation, the control of crystal shape, the inhibition of crystal growth and simply act as a template for depositing crystals [7]. It has been demonstrated that many proteins involved in biomineralization have an unusual amino acid composition with a high content of acidic residues [8]. Many of them also exhibit an IDP-like character or are simply IDPs [9], a class of proteins which do not form rigid 3D structures under physiological conditions, either along their entire length or only in localized regions [10], [11], [60]. AP7 is a mollusk shell IDP. The N-terminus of AP7 is a calcite binging, random-coil region, which affects calcium carbonate crystal growth *in vitro*, while C-terminal, α-helical region does not affect crystals growth [76]–[78]. Another protein from the mollusk shell is n16. In solution n16 exhibits random-coil structure, however increasing concentration of peptide as well as interaction with chitin cause disordered to ordered transition [79], [80]. CAP-1 is a protein responsible for exoskeleton formation of *Procambrus clarkia* [81]. It has been shown that the N-terminal fragment of CAP-1 inhibits crystal growth less efficiently, because it has more ordered structure than C-terminal fragment which is disordered [82], [83].

The recently-identified medaka *stm-l* gene is a putative homologue of the zebrafish *stm* gene [15]. Although there is no significant sequence similarity between *stm-l* and *stm* encoded proteins, it was previously suggested that Stm-l could be involved in the biomineralization of otoliths as was described earlier for Stm [13]. However, the protein encoded by *stm-l* gene has not been characterized yet. We aimed to characterize the structural properties of Stm-l, to lay the foundation for systematic studies on this protein involved in molecular mechanism of calcium carbonate biomineralization. We have elaborated and optimized a protocol for the efficient expression and purification of homogeneous, non-tagged Stm-l, and using a diverse array of biochemical, biophysical and bioinformatic methods, we carried out the first structural characterization of Stm-l. In this paper we demonstrated that Stm-l appears to have properties that are characteristic of an IDP with a tendency to form a locally ordered structure. Moreover, we elaborated an experiment which showed that Stm-l affects calcium carbonate biomineralization *in vitro*.

An examination of the amino acid composition of Stm-l showed that it was deficient in order-promoting amino acid residues and at the same time abundant in residues characterized as disorder-promoting, which is consistent with other IDPs (Fig. 2A). Stm-l can be classified as a very acidic protein with a high content of aspartyl and glutamyl residues, 16.9% and 20.4%, respectively, which predispose it to bind a large number of calcium ions. It also has a number of basic residues, 10.0%, that could be sites that enable interaction with carbonate counter ions. These features may play a crucial role in calcium carbonate biomineralization. Interestingly, in the primary Stm-l sequence, there are many EE motifs (16 repeats) and the content of E residues is higher than of D residues – something that is rather rare in proteins associated with calcium carbonate biominerals [8] (S1 Figure). The carboxylation of E residues is one of the post-translational modifications of the protein and leads to the formation of γ-carboxyglutamic acid. It was previously shown that some proteins containing such modified residues, like osteocalcin, are important constituents of calcium phosphate biominerals [84]. However, it should be pointed out that γ-carboxyglutamic acid has not yet been detected in proteins involved in calcium carbonate biomineralization [8]. Stm-l also contains many S residues, a total of 55 or 15.2%. Bioinformatic tools NetPhos 2.0 [85] and Disphos 1.3 [86], [87] predict that almost all these residues could be phosphorylated, 95% using NetPhos 2.0, and 96% using Disphos 1.3 (data not shown). Proteins involved in biomineralization are often highly phosphorylated, which is also associated with the disordered nature of these proteins. The lack of a hydrophobic core and rapid fluctuations in the peptide chain are known to expose a larger number of potential phosphorylation sites and lead to significantly greater phosphorylation than in the case of globular proteins [87]. This post-translational modification significantly affects biological functioning. It is noteworthy, that phosphorylation has a significant impact on the structure of DSPP, a human homolog of Stm-l [88]. There are some well-documented examples which have shown that the binding ability of counter ions might dramatically change when a protein is post-translationally modified [88], [89]. However, there are some examples, like osteopontin (OPN), where phosphorylation did not affect the Mg^2+^ and Ca^2+^ binding capacity of protein in solution [90]. It was suggested that counter ions occupy the majority of the acidic residue within OPN, whereas free phosphate groups are engaged in the crystal surface binding [90]. According to Wojtas et al. [40], the phosphorylation of Stm, the putative functional analog of Stm-l, increases its ability to control the formation of calcium carbonate crystals. Because our expression and purification procedure resulted in a protein that was completely devoid of post-translational modifications, further research steps could be to analyze of the possible role of post-translational modifications on the structure and function of Stm-l.

An analysis of the amino acid composition revealed additional interesting features of the Stm-l sequences. The high content of M residues in the Stm-l sequence, a total number of 11, is surprising because of the order-promoting character of this residue, which is rather hydrophobic and usually embedded inside the protein structure. It was previously shown that surface-exposed M residues could be involved in important functions such as antioxidant defense [91], [92] and the regulation of protein functions [92], [93] by reversible oxidation and reduction. Functional changes from the oxidation of M to methionine sulfoxide in a given protein appear to have pathophysiological significance in some cases [94]–[96]. A change in the hydrophobic M residue to the less hydrophobic methionine sulfoxide also has an impact on the structure of a protein [93], [95]. In addition, the oxidation of M residues increases the susceptibility of some proteins to ligation with ubiquitin and targets them for proteolytic degradation by the proteasome [97]. It is not clear at this time which of these functions M could fulfill in Stm-l, but this fact seems to be worthy of attention. The total protein concentration in the endolymph, a large acellular compartment where the biomineralization of otoliths takes place, was observed to change cyclically [98], [99]. Concentration fluctuations were postulated to be caused by the cyclical secretion/synthesis activity of the saccular epithelium associated with endolymphatic proteolysis [100]. If the oxidation of M residues increases the proteolysis susceptibility of Stm-l, it may play an important role in the spatial and temporal regulation of the prolificacy of Stm-l *in vivo*.

Predictions about the propensity of Stm-l for being disordered were generally consistent when carried out using different bioinformatics tools (Fig. 2C). The data clearly classified Stm-l as an IDP. However, some potential regions of order were predicted by PONDR-VLXT and the NPS algorithm, and three of these secondary structural elements were predicted by both tools (Fig. 2C and D). PONDR-FIT is a meta-predictor that combines six individual predictors, including PONDR-VLXT. PONDR-FIT has been shown to be somewhat more accurate than each of the component predictors [22] and for this reason it is a more reliable tool for predicting disorder. PONDR-VLXT is also very sensitive to local compositional bias and is capable of identifying potential interaction motifs, or molecular recognition features (MoRFs). Potential binding regions able to fold into α-helices upon binding to specific partners (α-MoRFs) are frequently observed as sharp dips flanked by long regions of predicted disorder in the PONDR-VLXT [101], [102]. All three potential MoRFs predicted by PONDR-VLXT for Stm-l (around residues 11–23, 255–269 and 323–340) are consistent with the NPS analysis, which shows the presence of α-helices in indicated parts of the Stm-l sequence.

The hydrodynamic behavior of Stm-l indicates that, in solution, it exists as a monomeric protein with a remarkably extended conformation, as evidenced by the unusually high R_S_ observed in the AUC and SEC experiments. In the far-UV CD experiments, although almost all of Stm-l was found to be unordered, a small amount of ordered secondary structures within the protein were observed (Table 3). Such residual structures are presumed to play a crucial role in the disorder-to-order transition of proteins and are involved in many molecular recognition events and binding to the physiological target of a given protein [103]–[105]. However, as was shown by the CD analysis, there was a low content of ordered structures, which is consistent with the SEC and CD experiments that showed there was no cooperative unfolding of Stm-l during GdmCl-induced denaturation. Low concentrations of GdmCl induced a significant decrease in the Stm-l R_S_, which suggests there was compaction of the protein (Fig. 4B). It has been shown that low concentrations of GdmCl can cause stabilization of a protein by eliminating the electrostatic repulsion of charged groups on the protein surface [106]. Because Stm-l possesses many acidic amino acid residues which could bind guanidinium ions, the observed compaction of Stm-l could have been induced by these ions. However, the CD spectra obtained for Stm-l at given concentrations of GdmCl indicated that this compaction did not correlate with an increase in the content of secondary structures (Fig. 4A). When concentrations of GdmCl were further increased, the stabilizing effect was overwhelmed by its own denaturing properties. Nevertheless, even at the highest GdmCl concentration, there was a slight change in the accessibility of Stm-l to the solvent compared to native conditions.

One of the characteristic features of IDPs is the ability to gain structure in response to variation in the environment [65]. We checked four agents that are known to induce conformational changes in IDPs: temperature, an organic solvent TFE, osmolyte TMAO, and counter ions. The analysis of temperature effects on the structural properties of Stm-l revealed that it exhibits the so-called turned out response to heat, characteristic for IDPs [65]. At low temperatures, Stm-l had a far-UV CD spectrum typical of an IDP. As the temperature was increased, the shape of the spectrum changed, reflecting the temperature-induced formation of a secondary structure. This temperature-induced folding was noncooperative and fully reversible (Fig. 5C). This is typical of coil-like IDPs and is attributed to the increased strength of the hydrophobic interactions at higher temperatures, which is the major driving force for folding [65]. Another sign of the folding propensity of Stm-l was provided by experiments where the CD spectra in increasing concentrations of TFE were recorded (Fig. 5A). Despite the fact that the shape and intensity of the CD spectra measured at different TMAO concentrations remained unchanged, we observed that in the presence of TFE, Stm-l underwent a conformational change which gave rise to an ordered structure, in this case α-helices. This could be consistent with the presence of the previously predicted α-MoRFs that bind to protein partners via disorder-to-order transitions resulting in an α-helix. Thus, our research showed that Stm-l is an extended coil-like IDP that is also very pliable with a tendency to form locally ordered structures.

The role of Stm-l in otolith formation has not been described yet. However, it has been suggested that Stm-l could be a functional analog of the zebrafish Stm [15], which appears to be involved in the organization of a protein scaffold of the otolith, and simultaneously in the intercalation of an organic matrix with calcium carbonate crystals [13], [40], [41], [69], [107]. In order to carry out these two functions, Stm-l would have to be capable of binding calcium ions and interacting with proteins. Studies conducted on calcium carbonate mineral interaction domains have identified that one of the key criterion that defines a calcium carbonate mineral recognition sequence is the presence of carboxylate residues [108], [109]. It is possible that the regular distribution of acidic amino acid residues in Stm-l (S1 Figure) serves as a template for binding calcium ions. In the series of SEC experiments, we demonstrated that an increase in the content of calcium ions was accompanied by essential changes in the R_S_ of Stm-l (Fig. 4D). The observed compaction of Stm-l caused by calcium ions constitutes indirect proof of these interactions. It is well known that counter ions are able to change the conformation of IDPs to a more compact structure by reducing the electrostatic repulsion between two regions of the protein [12], [65]. However, the far-UV CD experiment indicated that calcium ions did not change the content of ordered secondary structures in Stm-l. Currently, there are two hypotheses describing the mechanisms of biomineral formation. One of them applies the classical crystal growth theory. This theory assumes that crystals grow from saturated solution, where the nucleation is initiated through reaching a critical size cluster, followed by the ion-by-ion addition to the surface. According to this mechanism organic molecules act as a template for crystal nucleation in order to modulate crystal growth in desired directions via specific organic-inorganic interactions [1], [2], [7]. Second hypothesis states that biominerals are formed by an amorphous precursor, where acidic proteins, as highly flexible polymers with ion-binding ability, induce the formation of the amorphous precursor and further stabilize it [110]. It was shown that at the early stages of calcium carbonate formation, a polymer-induced liquid-precursor (PILP) is formed, which exists as a highly hydrated phase even more labile than the amorphous phase [111]. Since disorder and flexibility of the protein might be an advantage for PILP formation, it is possible that the disorder-to-order transition of Stm-l does not occur during the crystal formation. Conformational instability is a common feature of proteins that bind to inorganic solids. Thus, proteins that belong to the family of IDPs have characteristic, which enable them to perform their functions in biomineralization processes [78], [112]. Their unusual acidic composition leads to strong electrostatic repulsion and results in an extended conformation of the protein, providing a much larger binding surface in comparison to the compact conformation of globular proteins. This is highly advantageous, especially when interactions occur with crystalline surfaces [108], [113]. We showed that Stm-l possesses the above-mentioned properties which could facilitate its interaction with the calcium carbonate crystals. It should be pointed out that the compaction of IDPs could be dependent on post-translational modifications. An example of such a situation might be DPP, one of the products from the proteolytic cleavage of DSPP. It was reported that phosphorylated DPP underwent a calcium-induced conformation change from an extended structure to a more compact one, but dephosphorylated DPP did not [114], [115]. Another good example is osteocalcin, which adopted a more ordered structure in the presence of calcium ions; however, these changes were significantly diminished when γ-carboxyglutamic acid residues were decarboxylated to E [116].

In both trout and turbot, spatial chemical investigation of the endolymph surrounding the otolith called the sagitta, the largest of the otoliths, showed a lack of uniformity, including in the Na^+^ and K^+^ concentrations. This was probably dependent on the activity of ion-transporting cells called ionocytes or mitochondrial rich cells [117]; therefore, we hypothesize that ionic strength could also have an impact on the properties of IDPs involved in biomineralization. Our hypothesis is supported by well-documented examples of ionic strength-dependent conformational changes in some proteins not involved in biomineralization [118], [119]. In our SEC experiments, we demonstrated that in the absence of NaCl, the R_S_ of Stm-l was significantly larger than in the presence of 150 mM of NaCl (Fig. 4D). At the highest calcium ion concentrations, the hydrodynamic properties of Stm-l were independent of the concentration of monovalent sodium ions. The Stokes radii were almost the same with and without NaCl and the calcium-induced conformational changes were definitely more pronounced in the absence of NaCl. Thus, the ionic strength of the environment is the next factor to exert an influence on the conformation of Stm-l and could also modulate the effect of Stm-l on calcium carbonate biomineralization.

An *in vitro* biomineralization assay indicated that Stm-l had a considerable impact on the size and morphology of calcium carbonate crystals (Fig. 6). The increased number of crystals suggests that Stm-l may affect the crystal nucleation. The highly charged character of Stm-l together with its potential calcium binding ability might lead to an increase in the local concentration of ions that could in turn facilitate the nucleation step. The ion-induced compaction of the Stm-l molecule will further increase the ion density. Although, neither PILP nor an amorphous phase were observed in our experiments, their presence at the early stages of the crystal formation cannot be excluded. It has been shown that such phases are usually formed after several hours [120]. It must be noticed that the amorphous phase is highly unstable and its presence might be highly dependent on the sample preparation procedures. In the case of Stm, PILP was observed on the crystal surface after 24 h of growth only when the crystals were not washed before SEM [40]. The impact on the morphology of calcium carbonate crystals seems to be dependent on the protein concentration. Crystals grown without the protein using the slow diffusion method [39] typically form calcite rhombohedra. When crystals were grown in the presence of Stm-l, there was a significant change in crystal morphology. At low concentrations of protein, we observed crystals with rounded edges, whereas further increases in the protein concentration caused the formation of characteristic stair-like structures. Similar effect we observed for crystals grown in the presence of Stm using our conditions in *in vitro* biomineralization test (data not shown). Based on the SEM images, we suggest that the effect of Stm-l on calcium carbonate mineralization could be also similar to that obtained for AP8 proteins isolated from aragonitic abalone shell nacre [121], [122]. In the presence of AP8 proteins, each rhombohedral face of the calcite crystals exhibited rounded acute edges, while the obtuse edges remained unmodified and straight [122]. We observed a similar result in crystals obtained in the presence of Stm-l. However, at the highest concentration of Stm-l, the obtuse edges were also slightly rounded. In both cases, in the presence of Stm-l or AP8 proteins, modified crystals were elongated along the c-axis and capped by rhombohedral faces [121]. Interestingly, there is no significant similarity between AP8 proteins and the Stm-l sequence. They are both unusually acidic [15], [122], which may be crucial for the interaction between the proteins and the surface of the crystal. It is also worth noting, that an increase in the protein concentration was connected with a decrease in the size of the crystals obtained. The decrease in the dimensions of the calcium carbonate crystals, depending on the protein concentration used, indicates that Stm-l acted as an inhibitor of crystal growth. Unfortunately, the molecular mechanism of the inhibitory activity of proteins on calcium carbonate mineralization is still unclear. It was previously suggested, that the inhibitory effect of proteins on crystal growth is related to the disordered molecular structure of the proteins [83], [113], [123]. It was reported, for example, that the flexibility of DPP facilitated the extension of the protein across the surface of a hydroxyapatite crystal and allowed it to cover the surface with only a small number of molecules, resulting in a highly inhibitory effect on crystal growth [113]. A different mechanism has been proposed for statherin, where solid-state NMR studies indicated that the N-terminal fragment of the protein had a helical conformation and strongly interacted with hydroxyapatite crystals, while the middle and C-terminal regions interacted weakly with crystals and were highly mobile. This mobility of statherin on the surface of a crystal could allow it to more effectively block nucleation sites than rigidly bound protein [123]. It is possible that Stm-l may act according to one of these models. However, additional experiments are needed to verify this hypothesis. Micro-Raman analysis of calcium carbonate crystals has shown that Stm-l either stabilizes calcite or does not affect crystal polymorph in our experimental conditions. Stm also promoted calcite growth *in vitro*, even though Söllner et al. [13] have shown that *in vivo* Stm is necessary to form aragonite otoliths. It is no so surprising, because endolimph contains many different ions and other proteins which also affect polymorph selection. Firstly, magnesium ions can promote aragonite formation [124], [125]. Secondly, there are many experiments which shown that simultaneous action of at least two components are required to switch calcite to less stable aragonite. Usually one of the factor is acidic protein and the other is scaffold molecule. Keene et al. [80] have shown that n16N protein can induce aragonite but only in the presence of β-chitin, otherwise it induces calcite.

## Conclusions

Biomineralization is a complex process involving a large number of macromolecules, yet there is still a lot of unknown information about how they affect and modulate crystal growth. We believe that the knowledge gained on the functioning of Stm-l could have direct implications in understanding the role of proteins in otolith biomineralization. Our experimental data showed that Stm-l is a highly disordered protein that exhibits a tendency to form locally ordered structures. Using various methods, including SEC and CD, we have demonstrated, that Stm-l is an extremely pliable protein able to adopt a more ordered and rigid structure as a result of different factors such as temperature, denaturants, TFE, and counter ions. Interestingly, these different factors induced different structural changes. We showed that temperature and TFE caused an increase in more ordered secondary structures, whereas a small concentration of denaturant and counter ions induced compaction of the protein that was not accompanied by the formation of additional secondary structures. Therefore, it is possible that different environmental factors may exhibit different effects on the conformation and function of Stm-l. We have demonstrated that Stm-l changed the morphology of calcium carbonate crystals *in vitro*. Although Stm-l inhibited crystal growth, the increased number of crystals in the presence of the protein might have been an indication that more nucleation sites were formed. The high content of negative residues in the Stm-l sequence might possibly have caused calcium ions from the solution to gather and promote crystal nucleation. The extended and pliable conformation of Stm-l may facilitate post-translational modifications of the protein, or facilitate its interaction with inorganic constituents of biominerals and other proteins in the organic matrix of otoliths. Thus, we can consider Stm-l to be a multifunctional protein capable of binding calcium ions and interacting with proteins.

## Supporting Information

Figure S1 **Amino acid sequence of Stm-l.** All acidic amino acids are highlighted in red.
(TIF)

Figure S2 **The effect of Stm-l on calcium carbonate mineralization.** (A) SEM images of calcium carbonate crystals grown 336 h. Crystals grown in the absence of any protein (a, h, o), in the presence of trypsin at a concentration of 100 µg/mL (b, i, p), and in the presence of Stm-l in the following concentrations: 1 µg/mL (c, j, q), 5 µg/mL (d, k, r), 10 µg/mL (e, l, s), 20 µg/mL (f, m, t), 50 µg/mL (g, n, u). Concentrations of calcium ions were 5 mM (a–g), 10 mM (h–n), or 20 mM (o–u). The scale bar on the upper left corner of each panel represents a 200 µm-distance. (B) 10× magnification of representative crystals shown on panel (A). The scale bar on the upper left corner of each panel represents a 20 µm-distance. Other details as in (A).
(TIF)
