# Correction: Intrinsically Disordered and Pliable Starmaker-Like Protein from Medaka (*Oryzias latipes*) Controls the Formation of Calcium Carbonate Crystals

**DOI:** 10.1371/journal.pone.0119969

**Published:** 2015-03-20

**Authors:** The PLOS ONE Staff

There are errors in the Author Contributions. The correct contributions are: Conceived and designed the experiments: MR AO MW MJ. Performed the experiments: MR MJ CS MG MM. Analyzed the data: MR AO MJ MM. Wrote the paper: MR AO MW.
