# Supplementary figures and images for: Intrinsically Disordered and Pliable Starmaker-Like Protein from Medaka (Oryzias latipes) Controls the Formation of Calcium Carbonate Crystals
Source: PLoS One. 2014 Dec 9;9(12):e114308. doi: 10.1371/journal.pone.0114308 (PMC4260845; doi:10.1371/journal.pone.0114308)

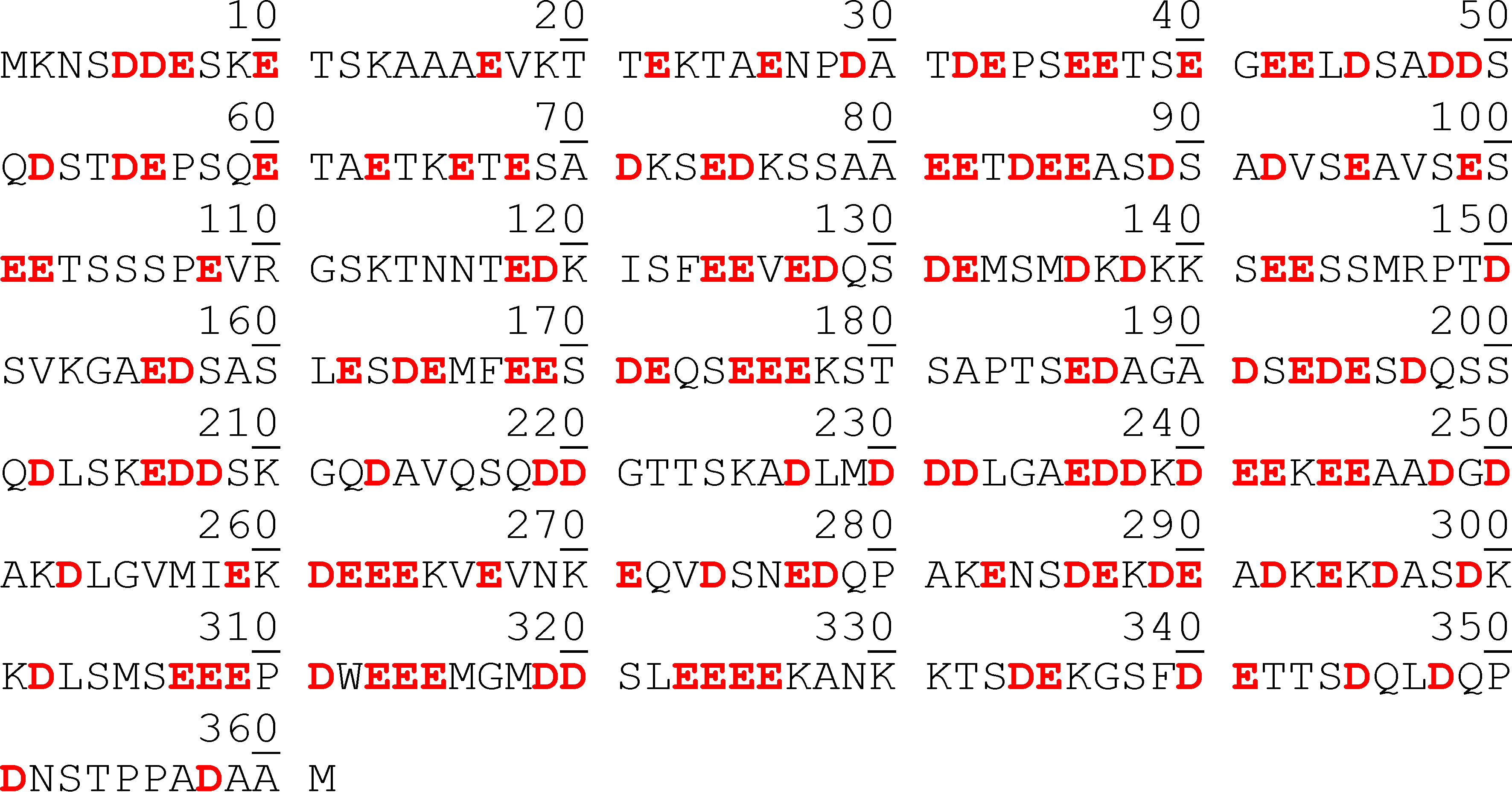

Supplement: Figure S1 — Amino acid sequence of Stm-l. All acidic amino acids are highlighted in red. (TIF) [file pone.0114308.s001.tif]

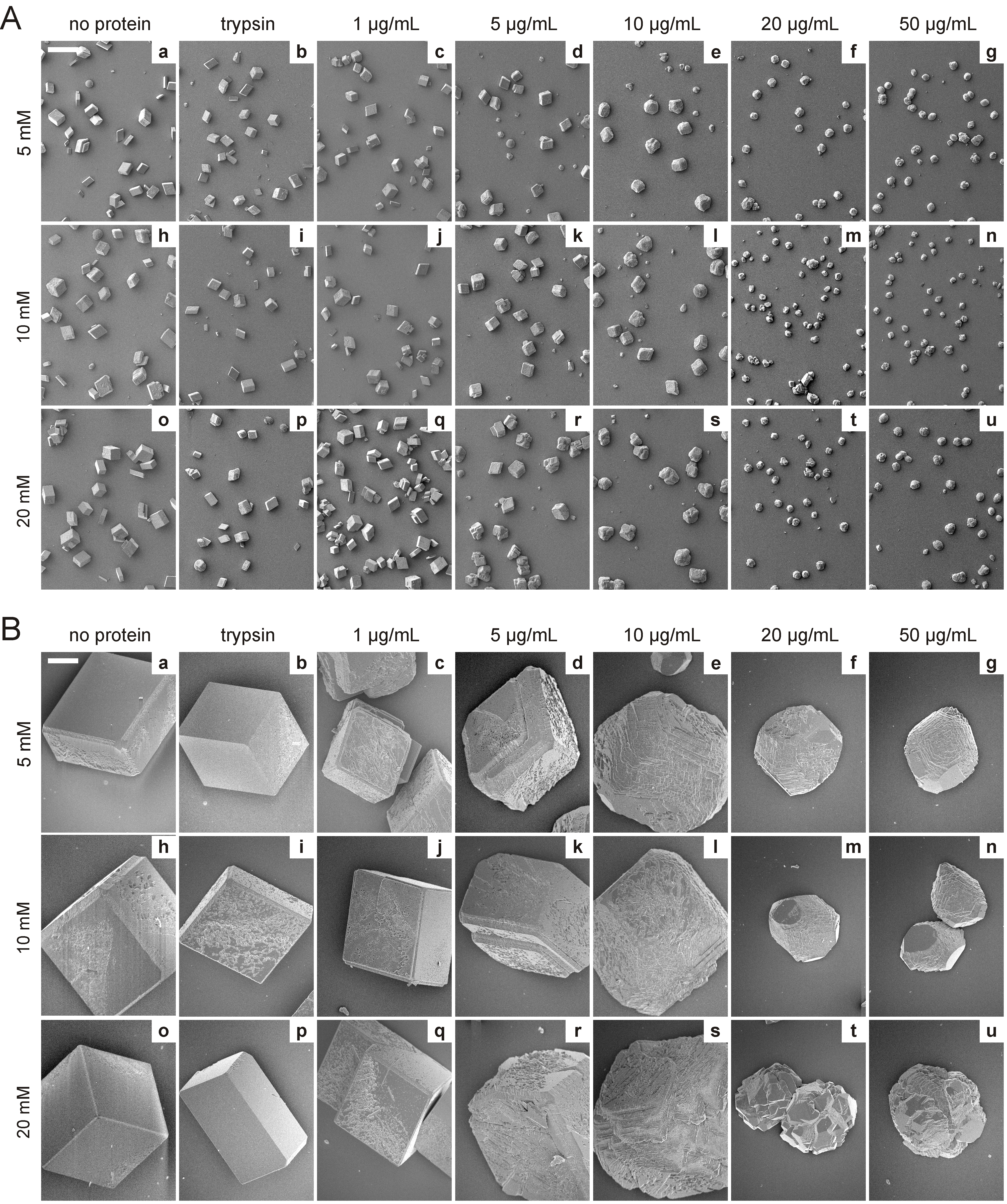

Supplement: Figure S2 — The effect of Stm-l on calcium carbonate mineralization. (A) SEM images of calcium carbonate crystals grown 336 h. Crystals grown in the absence of any protein (a, h, o), in the presence of trypsin at a concentration of 100 µg/mL (b, i, p), and in the presence of Stm-l in the following concentrations: 1 µg/mL (c, j, q), 5 µg/mL (d, k, r), 10 µg/mL (e, l, s), 20 µg/mL (f, m, t), 50 µg/mL (g, n, u). Concentrations of calcium ions were 5 mM (a–g), 10 mM (h–n), or 20 mM (o–u). The scale bar on the upper left corner of each panel represents a 200 µm-distance. (B) 10× magnification of representative crystals shown on panel (A). The scale bar on the upper left corner of each panel represents a 20 µm-distance. Other details as in (A). (TIF) [file pone.0114308.s002.tif]
